# Royal Medical and Chirurgical Society

**Published:** 1896-11-14

**Authors:** 


### ROYAL MEDICAL AND CHIRURGICAL SOCIETY.

At the meeting held on November 10th, a very interesting case of locomotor ataxy, occurring in a young woman, showing tabetic arthropathy with dislocation of both hips, and in which six pregnancies had occurred in the course of the disease, was described by Dr. Thomas Wilson, of Birmingham.

The disease began at the age of sixteen with shooting pains, especially felt in the lower extremities. The patient was married at the age of twenty-three, and had six pregnancies, the fifth ending in a miscarriage at the fifth month, the other five in the birth of living childen at full term. Arthropathy of the knees and hips began at twenty-four, at the time of the second delivery, and dislocation of the hips took place at twenty-six, after the birth of the third child. The symptoms of tabes, other than the joint affection, included unsteadiness on standing with the eyes shut, athetoid movements, loss of knee-jerks, impairment of tactile, painful, and thermal sensation, loss of muscular sense, spontaneous fracture of the ileum, temporary strabismus, Agryll-Roberson pupils, pains of various kinds, and some affection of the sphincters. There was no proof of syphilis, acquired or inherited. There was a strong family tendency to nerve degeneration, as shown by a paralysis in a paternal aunt, who was never able to feed herself or speak, and died at the age of twenty-three, by the suicide of one brother, and by the attempted suicide of another.

As far as could be ascertained, the progress of the nervous affection was not notably influenced by the repeated pregnancies; the tabetic symptoms increased rather rapidly from the time of the second delivery onwards for about two years, after which they became stationary, and remained so for the whole of the last two years, during which the woman had been under observation; two of the six pregnancies had taken place in these last two years.

